# Local Treatment of Non-small Cell Lung Cancer with a Spray-Dried Bevacizumab Formulation

**DOI:** 10.1208/s12249-021-02095-7

**Published:** 2021-08-31

**Authors:** Kimberly B. Shepard, David T. Vodak, Philip J. Kuehl, David Revelli, Yue Zhou, Amanda M. Pluntze, Molly S. Adam, Julia C. Oddo, Lauren Switala, Jonathan L. Cape, John M. Baumann, Michael Banks

**Affiliations:** 1 Research & Development, Lonza, 64550 Research Rd., Bend, Oregon, 97703 USA; 2 Lovelace Biomedical, Albuquerque, New Mexico USA; 3 Global Business Development, Lonza, Portsmouth, New Hampshire USA

**Keywords:** spray drying, monoclonal antibody, biotherapeutics, lung cancer, local delivery

## Abstract

Local delivery of biotherapeutics to the lung holds great promise for treatment of lung diseases, but development of physically stable, biologically active dry powder formulations of large molecules for inhalation has remained a challenge. Here, spray drying was used to manufacture a dry powder pulmonary formulation of bevacizumab, a monoclonal antibody approved to treat non-small cell lung cancer (NSCLC) by intravenous infusion. By reformulating bevacizumab for local delivery, reduced side effects, lower doses, and improved patient compliance are possible. The formulation had aerosol properties suitable for delivery to the deep lung, as well as good physical stability at ambient temperature for at least 6 months. Bevacizumab’s anti-VEGF bioactivity was not impacted by the manufacturing process. The formulation was efficacious in an in vivo rat model for NSCLC at a 10-fold decrease in dose relative to the intravenous control.

## Introduction

Noninvasive strategies to deliver biologic active pharmaceutical ingredients (APIs), such as proteins, peptides, and antibodies, have generated intense interest [1]. For lung indications, local treatment by delivering inhaled formulations directly to the site of action, when appropriate, is preferred, since this approach avoids the drawbacks of systemic delivery, making reduced side effects, lower doses, convenient at-home administration, and improved patient compliance possible. Local treatment of lung diseases is common for indications such as asthma and chronic obstructive pulmonary disease (COPD), with more than 100 inhalation products approved by the US Food and Drug Administration (FDA) on the market as of 2018 [2]. Here, we explore expansion of the local delivery concept for lung cancer treatment using a spray-dried monoclonal antibody (mAb) delivered with a dry powder inhaler.

As of 2017, lung cancer was the leading cause of cancer-related deaths in Americans, with non-small cell lung cancer (NSCLC) making up the majority of cases [3]. Lung cancer is treated with a combination of small molecule chemotherapeutics, radiation, and biotherapeutics. Delivery directly to the lung has been proposed as a means to avoid systemic toxicity and side effects [4–6]. In previous studies with small molecule APIs, local treatment of a preclinical lung cancer model using dry powder inhalers has proven efficacious [7,8]. An extension of this approach to biotherapeutic lung cancer APIs such as bevacizumab could present numerous benefits.

Bevacizumab is a recombinant humanized mAb that acts as an anti-angiogenic agent. It is used to treat lung cancer, colon cancer, glioblastoma, renal cell carcinoma, and age-related macular degeneration. Bevacizumab reduces tumor growth by interrupting formation of new blood vessels. Specifically, it disrupts the vascular endothelial growth factor (VEGF) pathway, which is critical in tumor vascularization [9]. A bevacizumab intravenous (IV) formulation has been approved for cancer treatment in combination with chemotherapy since 2004.

For advanced NSCLC, bevacizumab is used as a first-line treatment with chemotherapeutic agents such as carboplatin or cisplatin [10,11]. Several clinical trials showed use of bevacizumab that significantly increased overall survival and progression-free survival in patients [12]. Bevacizumab treatment is often continued as a maintenance therapy after the patient can no longer tolerate chemotherapy to reduce regrowth of tumors [13,14].

However, numerous challenges are associated with bevacizumab therapy because it is delivered systemically. The therapy is expensive and must be administered in a clinical setting by IV infusion, typically at 7.5 to 15 mg/kg [13,14]. Due to this high dose and challenges with concentrating mAb solutions above 50 mg/mL, intramuscular or subcutaneous injections are not feasible. Maintenance treatment with bevacizumab is typically delivered by IV infusion every 3 weeks until the disease progresses—a regimen that is expensive, inconvenient, and time-consuming, testing patient compliance [15].

Because of the high bevacizumab doses that are required and the lack of specificity associated with systemic treatment, many patients must be excluded from treatment due to the risk of serious adverse effects including pulmonary hemorrhage. To reduce the risk of serious bleeding, many patients are excluded from treatment for any of the following reasons: squamous histology, age over 75, bleeding in the airway, brain metastases, or tumors near or inside major blood vessels [3].

Local delivery of bevacizumab to the site of the tumor (*e.g.*, the lung for NSCLC) would reduce the risk of adverse events and enable use of a much smaller dose because delivery is targeted, reducing systemic exposure. This approach could reduce side effects and adverse events, potentially enabling treatment for a wider range of patients. Local delivery would also be more convenient for patients, since an inhaled formulation would allow self-administration of the maintenance treatment, reducing cost and improving patient compliance.

Development of inhaled formulations to deliver large, delicate molecules such as mAbs has proven challenging, but success has been achieved using spray drying to generate respirable powders of small molecules for drug delivery to the deep lung [8,16–20]. In spray drying, the API and excipients are co-dissolved in a solvent. The resulting solution is pumped into a drying chamber, where it is atomized into droplets. The droplets come into contact with drying gas, which rapidly removes the solvent, forming solid microparticles. By fine-tuning the spray drying process parameters, powders suitable for inhaled delivery (*e.g.*, with aerodynamic diameters < 5 μm) can be generated. Spray drying of proteins and mAbs has been demonstrated in the literature, typically for reconstitution into an IV formulation, and a few reports for inhalation [21–23]. A recent review extensively detailed work on spray-dried proteins [24].

This work demonstrates the successful development of a spray-dried bevacizumab formulation with preserved biological activity, good physical stability, and the physico-chemical and aerosol performance characteristics needed for inhaled delivery. The inhaled bevacizumab formulation was tested for efficacy in vivo using an orthotopic lung cancer model in nude rats, where it had comparable efficacy to an injected bevacizumab formulation at one-tenth the IV dose.

## Materials and Methods

### Materials

Bevacizumab drug substance was supplied as a sterile solution of 30 mg/mL bevacizumab in 50 mM phosphate buffer, pH 6.2, with 60 mg/mL trehalose and 0.04% polysorbate 20. Trehalose dihydrate was purchased from Pfanstiehl (Waukegan, IL, USA), and L-leucine was purchased from J.T. Baker Inc. (Phillipsburg, NJ, USA).

### Methods

#### Spray Drying

Bevacizumab solution was used as received. It was placed inside a Snakeskin dialysis membrane (10,000 Dalton molecular weight cutoff) (Thermo Fisher Scientific Co., Waltham, MA, USA) and clipped on both ends. The membrane was floated in 1 mM sodium phosphate buffer with 20 mg/mL trehalose, at a volume ratio of 1:100, and gently stirred. Dialysis lasted 24 h with one buffer replacement.

Spray drying was conducted on a custom laboratory scale dryer with a nominal drying gas flow rate of 35 kg/h nitrogen. The liquid spray solution was fed to the dryer using a peristaltic pump and atomized through a two-fluid nozzle (Model ¼ J, with a 1650 liquid body and 64 air cap, Spraying Systems Co., Wheaton, IL, USA). The outlet temperature was 50°C. A 2-inch cyclone was used to collect the powder in a glass jar. The powder was then dried under vacuum at ambient temperature with a nitrogen sweep gas and stored with desiccant at 5°C. The resulting spray-dried particle had a target morphology of an amorphous phase containing trehalose and bevacizumab, with crystalline L-leucine enriched on the particle surface to act as a dispersing agent.

#### Powder X-Ray Diffraction (PXRD)

To assess the crystallinity of the L-leucine in the spray-dried powder, PXRD patterns were collected using a MiniFlex 600 instrument (Rigaku Corporation, Tokyo, Japan) using a copper anode generator (*K*_α1_ = 1.54060 Å; *K*_α2_ = 1.54439 Å, 45 kV, 15 mA). Samples were placed on a zero-background sample cup and analyzed over a 2Θ range of 3 to 40°, at a rate of 2.5° 2 Θ/min. As-received L-leucine was used as a reference material to compare with the polymorph found in the spray-dried powder.

#### Differential Scanning Calorimetry (DSC)

DSC was performed using a DSC3+ instrument (Mettler Toledo, Columbus, OH, USA). To measure the glass-transition temperature (*T*_g_) of the amorphous material in the spray-dried powder, samples were sealed in aluminum pans, vented, and scanned in ADSC mode (a single-frequency temperature-modulated DSC technique) from 0 to 170°C at 2.5°C/min with a modulation of 1.5°C every 60 s. The *T*_g_ was analyzed using STAR^e^ software (Mettler Toledo), reporting the onset and midpoint temperatures of the transition. To measure the unfolding (melting) temperature of the as-received bevacizumab in solution, 10 μL of solution was pipetted into a 40-μL aluminum pan and hermetically sealed. Then 10 μL of pH 6.3 phosphate buffer was pipetted into the reference pan to subtract the contribution of the liquid to the thermal trace. The sample was scanned from 30 to 110°C at 5°C/min.

#### Karl Fischer (KF) Titration

The water content of the spray-dried powder was measured using a coulometric Metrohm® 851 Titrando KF oven titrator (Metrohm USA Inc., Tampa, FL, USA), with the generator electrode operated in diaphragm-less mode. A 10- to 30-mg sample was sealed into a crimped KF vial and analyzed at 105°C.

#### Scanning Electron Microscopy (SEM)

To assess morphology, SEM images of bevacizumab spray-dried powders were obtained using a Hitachi SU3500 (Hitachi High Technologies America Inc., Schaumburg, IL, USA). A trace amount of sample was applied to double-sided carbon tape mounted on an aluminum stub. The sample was then sputter-coated with gold/palladium for 10 min at 15 to 20 mV using a Hummer® 6.2 Sputter System (Anatech Ltd., Battle Creek, MI, USA).

#### Size-Exclusion Chromatography with Multiple-Angle Laser Light Scattering (SEC-MALLS)

Bevacizumab spray-dried powder and control solution was analyzed by SEC-MALLS to determine the presence of high molecular weight species (*e.g.*, dimers and trimers of the mAb). Materials were diluted to 5 mg/mL with pH 6.3 phosphate buffer. An Agilent 1100 high-performance liquid chromatography (HPLC) instrument (Agilent Technologies, Santa Clara, CA, USA) was used with a TSKgel GMPW_XL_ column (7.8 mm ID, 30-cm length, 13-μm particle size, 10- to 100-nm pore size)(Tosoh Bioscience, Tokyo, Japan). The mobile phase was pH 7.4 phosphate buffered saline. Samples were run isocratically at a flow rate of 0.8 mL/min for 50 min with an injection volume of 20 μL.

#### Geometric Particle Size Distribution

The geometric particle size distribution of the spray-dried powder was measured with a Malvern Mastersizer 3000 using an Aero S dry powder disperser. Mastersizer software was used to analyze the results using the Fraunhofer approximation. Samples were run in triplicate with obscuration levels between 0.1 and 8%, disperser pressure of 2 to 3 bar, and feed rate of 30 to 70%.

#### Aerodynamic Particle Size Distribution

The aerodynamic particle size distribution of spray-dried powder was measured using a TSI Aerodynamic Particle Sizer® 3321 spectrometer with a Model 3433 small-scale powder disperser and Model 3302A diluter (TSI, Shoreview, MN, USA). The air flow rate was 18.5 L/min in the powder disperser, and the sheath flow rate was 4 L/min. The diluter used a 100:1 capillary at pressure of 0.32 in. of water. Samples were measured in triplicate for 30 s each.

#### Next Generation Impactor (NGI)

The aerosolization properties of the spray-dried powder were analyzed using an MSP NGI Model 170, MSP Corp., Shoreview, MN, USA) with a high-resistance 4-kPa Plastiape dry powder inhaler (Plastiape S.p.a., Osnago, Italy). Spray-dried powder (10 mg) was hand-filled into size 3 Vcaps® Plus capsules (Lonza, Morristown, NJ, USA). A pre-separator containing 10 mL of pH 7.4 PBS was used upstream of the NGI. The test was operated at 65 L/min for 4.0 s. The contents of Pans 2 through 7 were dissolved in 5 mL of pH 7.4 PBS, and Pans 1 and 8 were dissolved in 10 mL of PBS. The bevacizumab content in the spray-dried powders was measured using an absorbance technique that employed ultraviolet (UV) probes (Pion Rainbow MicroDISS Profiler™, 20-mm path length). Standards were prepared using as-received bevacizumab stock. The second derivative of the absorbance over the range from 276 to 284 nm was used to quantify the bevacizumab, because trehalose and L-leucine do not absorb at this wavelength range.

#### Activity Assay

A reporter-based assay (Promega, Madison, WI, USA) for anti-VEGF antibodies was used to determine the biological activity of the spray-dried powders. Detailed information about the assay is available on Promega’s website and in Wang et al. [25]. In the VEGF bioassay, an engineered cell line (KDF/NFAT-RE HEK293) that expresses VEGF receptor-2 (VEGFR2/KDR) was used, combined with a VEGF-stimulated luciferase reporter. When VEGF binds to KDR, luminescence is induced, which is detected by adding the kit’s Bio-Glo™ reagent and quantified with a luminometer. When the anti-VEGF antibody is present, VEGF has reduced or no binding to KDR, and luminescence is reduced.

#### Formulation Selection

Spray-dried powders with three active loadings were manufactured for the feasibility stage of this study, consisting of 10/70/20, 20/60/20, and 40/40/20 bevacizumab/trehalose/L-leucine (by weight). (For brevity, these will be referred to as the 10%, 20%, and 40% formulations.) The physical stability, aerosol properties, and biological activity of the formulations were evaluated. For physical stability, we focused on two characteristics: (1) the L-leucine portion of the spray-dried powder should be crystalline, and (2) the amorphous phase containing trehalose and bevacizumab should have a high-onset glass-transition temperature (*T*_g_).

All three formulations met these criteria, exhibiting *T*_g_ onset temperatures of ~117°C and PXRD peaks that were characteristic of spray-dried crystalline L-leucine. The aerosol properties of the formulations, measured by NGI, are shown in Table I. The 10% and 40% formulations met the MMAD specification of 2 to 3 μm, and the 40% formulation had the highest FPF. Biological activity was assessed using the anti-VEGF activity assay described above. All three formulations inhibited VEGF expression similar to that of a bevacizumab solution control.

**Table I:** Analytical Results for Bevacizumab Feasibility Formulations Before and After Storage for 2 Weeks at 40°C/75% RH

**Condition**^**a**^	**Value**					
	**10% Formulation**		**20% Formulation**		**40% Formulation**	
	**Initial**	**2 wk**	**Initial**	**2 wk**	**Initial**	**2 wk**
Onset *T*_g_ (°C)	117	117	117	117	117	117
MMAD (μm)	2.4	2.1	1.6	2.1	2.4	2.5
FPF (%)	66	72	73	74	78	75
VEGF activity assay (IC_50_/IC_50, control_)	0.93	1.03	1.26	1.38	0.97	1.08

^a^*MMAD* median mass aerodynamic diameter, *FPF* fine-particle fraction (defined here as the mass percentage of drug particles with an aerodynamic diameter <5 μm), *IC*_*50*_ concentration of a drug that reduces the luminescense by 50%

The three formulations were also subjected to an accelerated stability challenge, where samples were stored for 2 weeks in a closed vial with desiccant at 40°C/75% relative humidity (RH). The same tests described above were repeated. The largest changes were found for the 20% formulation, and only small changes were observed for 10% and 40% formulations. Based on these results, the 40% bevacizumab spray-dried powder was selected as the lead formulation for the remainder of this study due to its good stability and high active loading. All further references to spray-dried powders refer to this formulation.

#### Real-Time Stability Study Design

A real-time stability study was conducted, storing the bevacizumab spray-dried powder at two conditions: 5°C and 25°C/60% RH. A sample of the spray-dried powder (150 mg) was sealed in a glass vial. Samples (10 mg each) were also filled into size 3 capsules (Vcaps Plus HPMC capsules, Capsugel) in triplicate for the 6-month stability sample and sealed in a glass vial. The vials were heat-sealed in a Mylar® bag containing 2 g of silica gel desiccant. Samples were removed for analysis after storage for 1, 3, and 6 months.

#### *In Vivo* Study Design

An *in vivo* study was designed for the bevacizumab spray-dried powder using an orthotopic nude rat model for NSCLC [26]. All protocols were reviewed and approved by an Institutional Animal Care and Use Committee (IACUC) at LBRI. Research was conducted under an IACUC-approved protocol in compliance with the Animal Welfare Act, PHS Policy, and other federal statutes and regulations relating to animals and experiments involving animals. The facilities where this research was conducted are accredited by the Association for Assessment and Accreditation of Laboratory Animal Care. The study tested the effect on tumor size for bevacizumab spray-dried powder delivered by inhalation (INH) and bevacizumab administered by intraperitoneal (IP) injection, with and without cisplatin, a chemotherapy medication. The NSCLC cell line Calu-3 was intratracheally instilled into the lungs of seven study groups of X-irradiated rats, targeting 1.5 × 10^7^ cells per installation [27]. No treatment was given for the first 4 weeks of the study, enabling growth of the tumor cells. The study design is shown in Table II.

**Table II:** *In Vivo* Study Design with NSCLC Orthotopic Nude Rat Model

**Study Group**	**Primary treatment (weeks 4–8)**		**Maintenance therapy (weeks 8–12)**^**a**^	**Rats**	**Endpoints**
	**Cisplatin**	**Bevacizumab**	**Bevacizumab**		
1^a^	No	No	No	15	• 8-week lung weight
2^b^	Yes (IP)	Yes (IP)	No	15	
3	No	Yes (INH)	No	15	
4	Yes (IP)	Yes (INH)	No	15	
5	No	No	No	20	• Survival • 12-week lung weight
6	Yes (IP)	Yes (IP)	Yes (INH)	15	
7	Yes (IP)	Yes (INH)	Yes (INH)	15	

^a^Negative control

^b^Positive control (standard of care for NSCLC)

During the primary treatment phase (weeks 4 through 8), bevacizumab was administered either by IP injection (15 mg/kg, once per week) or by INH (15 mg/kg presented dose, 1.5 mg/kg deposited dose, once per week). For some study groups, cisplatin was administered by IP injection (3 mg/kg). For INH administration, the spray-dried powder was aerosolized using a rotating-brush generator and delivered to the rats passively through nasal inhalation. During the maintenance treatment phase (weeks 8 through 12), only INH bevacizumab was administered (15 mg/kg presented dose, 1.5 mg/kg deposited dose, once per week). No additional cisplatin was administered. Groups 1 through 4 were evaluated for primary efficacy after 8 weeks with lung weight as the endpoint. Groups 5 through 7 were evaluated for maintenance efficacy after 12 weeks with lung weight and survival as the endpoints.

## Results

### Manufacturing of 40% Bevacizumab Spray-Dried Formulation

#### Solution Thermal Stability

During spray drying, particles encounter temperatures ranging from the wet-bulb temperature of the environment to the outlet temperature of the dryer. To ensure thermal stability, the unfolding (melting) temperature of bevacizumab in solution was quantified using DSC. Two endothermic peaks were observed, in qualitative agreement from literature reports [28]. The onset of the lowest peak occurred at 71°C. In a report from Akbas et al. [29], DLS was used to measure aggregate formation in bevacizumab solutions as a function of temperature. In this study, large aggregates were detectable by DLS starting at 61°C. Therefore, the upper limit to the spray dryer outlet temperature was set at ~55°C.

#### Spray Drying

Spray drying a biologic material such as bevacizumab for inhalation applications requires careful selection of process conditions. First, droplet size must be controlled to generate a respirable particle with a target aerodynamic diameter of <5 μm. The spray solution solids loading, atomization conditions, and drying kinetics can all impact the resulting aerodynamic diameter of the product. Second, to reduce degradation of the thermally labile biologic material, temperature exposure must be limited. This is a balancing act, since the drying process must still rapidly remove enough water from the product that the trehalose remains amorphous and good yield is achieved. To this end, an outlet temperature of 50°C was chosen, and the drying gas flow rate was maximized for the spray dryer geometry. A yield of 90%, including residual water, was observed for the 40-g batch of spray-dried powder prepared for the *in vivo* study.

### Physical State of Bevacizumab Spray-Dried Powder

#### Water Content

The water content of the spray-dried powder is important to ensure stability, good aerosol properties, and powder flow. Spray-dried powders with adequate physical stability should retain their physical state for at least 2 years without recrystallization of the amorphous phase. Amorphous trehalose is known to recrystallize to its dihydrate form if the water content is too high, resulting in destabilization. However, formulations with water contents below 1 to 2% may result in static issues, reducing aerosol performance. To this end, the water content of the spray-dried powder was evaluated after storage with desiccant for 72 h by Karl Fisher titration and was 3 to 4% (by weight)—low enough to prevent recrystallization of the amorphous trehalose while maintaining acceptable aerosol properties.

#### Leucine Crystallinity

**Figure 1:**
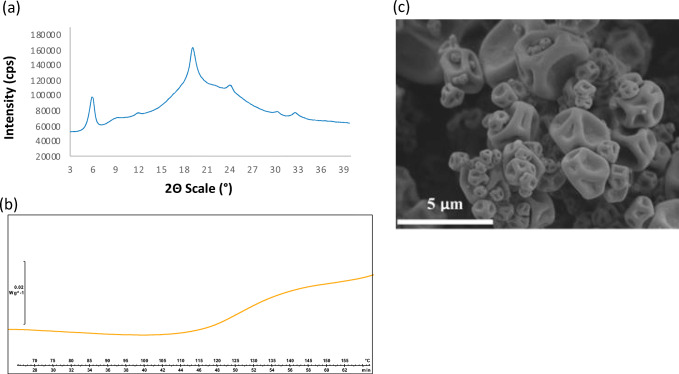
Characterization results for bevacizumab spray-dried powder, showing PXRD diffractogram (**a**), reversing heating capacity by DSC (**b**), and SEM image (**c**)

By design, the bevacizumab spray-dried powder consists of two phases: crystalline L-leucine and an amorphous phase of trehalose and bevacizumab. Vehring and coworkers have demonstrated that L-leucine must enrich and crystallize at the surface of the droplet during spray drying to maximize L-leucine’s performance as a dispersing agent [30–33]. PXRD analysis was conducted on the bevacizumab spray-dried powder to qualitatively determine whether L-leucine is crystalline. As Figure 1a shows, the characteristic peaks of spray-dried crystalline L-leucine were observed. The diffractogram of the spray-dried crystalline L-leucine did not exactly match that of the as-received crystalline L-leucine, but this phenomenon has been reported elsewhere [34,35] and is likely due to the submicron-sized crystalline domains formed during rapid spray drying. No peaks characteristic of trehalose dihydrate were observed. Superimposed on the characteristic L-leucine peaks was the amorphous halo characteristic of the trehalose/bevacizumab phase.

#### Thermal Analysis

The *T*_g_ of an amorphous material is a useful metric for estimating physical stability after storage. To minimize molecular mobility on a timescale of years, the *T*_g_ should ideally be ~50°C higher than the storage temperature of the material [36]. The thermal properties of the bevacizumab spray-dried powder were measured by DSC. As the results in Figure 1b show, no melt peaks were observed in the DSC trace. Crystalline L-leucine has no expected thermal transitions in the range of temperatures scanned. A broad *T*_g_ was observed in the reversing heat flow trace with an onset temperature of 117°C and a midpoint temperature of 128°C. This confirmed that trehalose and bevacizumab form a homogeneous amorphous phase in the spray-dried powder.

The bevacizumab had an anti-plasticizing effect on the trehalose, raising the *T*_g_ of the amorphous material (pure trehalose has an onset temperature of 106°C [37]), suggesting an interaction between the two components, such as hydrogen bonding. This effect has been previously observed in amorphous solid dispersions [38]. No thermal signature of degradation was observed in the DSC trace until 155°C, at which point trehalose begins to decompose as well. Overall, thermal analysis indicated that the material has a low risk of failure during storage, as the *T*_g_ is nearly 100°C above its intended storage temperature.

### Aerosol Performance of Bevacizumab Spray-Dried Powder

#### Morphology

The morphology of the bevacizumab spray-dried powder was observed using SEM. As the representative image in Figure 1c shows, no evidence of particle fusion was observed. Collapsed spherical particles were formed during spray drying, with most particles ~1 to 5 μm in diameter.

#### Particle Size Distribution

The geometric and aerodynamic particle size distributions of the spray-dried powder were measured using light scattering and aerodynamic particle sizing by time-of-flight laser velocimetry, respectively. For geometric particle size, the Sauter mean diameter (*D*_32_) was 2.3 μm, *D*_10_ = 0.3 μm, *D*_50_ = 2.2 μm, and *D*_90_ = 4.4 μm. For aerodynamic particle size, the MMAD was 2.0 μm with a geometric standard deviation of 1.6 μm. For spherical particles, the aerodynamic particle size is equal to the geometric particle size times the square root of the particle density. By comparing these distributions, we found that the particle density was approximately 0.8 to 1.0 g/cc, implying the presence of void volume inside the particle.

#### NGI

**Figure 2.**
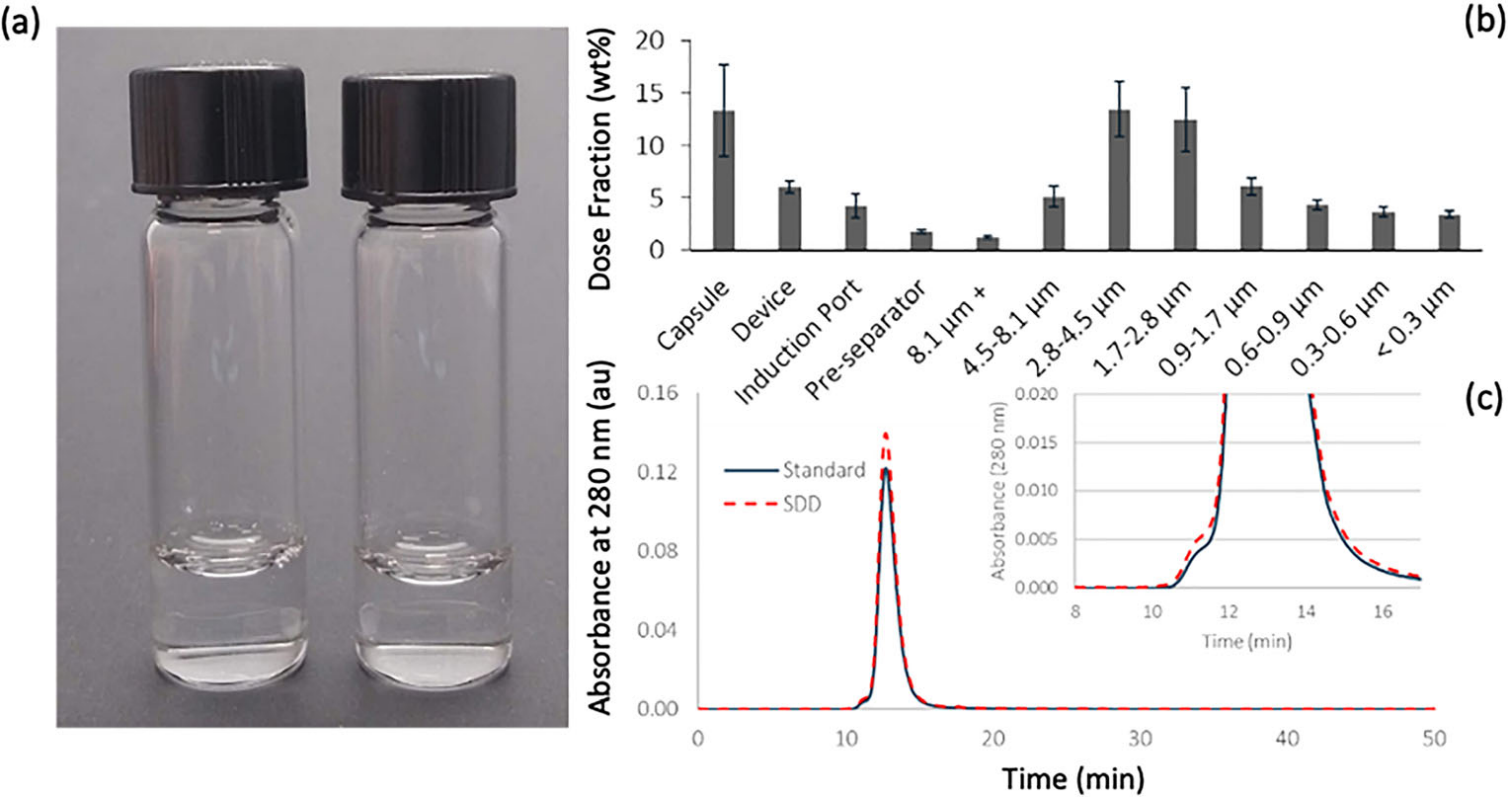
Photo of reconstituted bevacizumab spray-dried powder in PBS (left) and spray-drying stock solution (right) (**a**), aerodynamic diameter distribution of bevacizumab spray-dried powder by NGI (**b**), SEC-MALLS chromatogram for reconstituted bevacizumab spray-dried powder (red dashes) and as-received bevacizumab solution (blue solid line) with inset showing low retention time shoulder corresponding to mAb dimers and trimers (**c**)

To ensure delivery to the deep lung, the fraction of powder with an aerodynamic diameter of 5 μm or less should be maximized. While the aerodynamic particle sizer measures the distribution of the powder when aggressively aerosolized, the NGI analyzes the distribution of powder emitted from a device under more biorelevant conditions. Using a high-resistance dry powder inhaler at a flow rate of 65 L/min on the NGI, most emitted particles were between 0.9 and 4.3 μm in aerodynamic diameter (Figure 2b). The FPF was 82%, and the MMAD was 2.2 μm. These results indicate favorable aerodynamic properties for delivery to the deep lung.

### Potency

The bevacizumab content of the spray-dried powder was confirmed using absorbance at 280 nm, with an average of 37% ± 1% by weight after adjustment for water content. This sub-potency compared to the 40% target was likely due small amounts of mAb binding to the in-line 0.1-μm filters used to ensure sterility of the spray solution. Any aggregates of bevacizumab created during the solution preparation and pumping process were retained in the filter, reducing the overall potency of the product.

### Aggregation of Reconstituted Bevacizumab Spray-Dried Powder

Bevacizumab spray-dried powder was reconstituted in buffer, resulting in an optically transparent solution, as shown in Figure 2a, with bevacizumab solution as-received as comparison. The solutions were also analyzed by SEC-MALLS, which is particularly sensitive to the presence of dimer and trimer aggregates. The control stock solution and reconstituted spray-dried powder showed similar quantities of aggregated species, which were small in comparison with the primary mAb peak (Figure 2c).

### Anti-VEGF Activity

Bevacizumab’s mechanism of action is inhibition of VEGF expression in cancer cells. To evaluate the material’s biologic activity before and after spray drying, a commercially available kit was used. The kit uses a VEGF-responsive reporter cell line to assay repression of the VEGF-induced activity by bevacizumab. The cell line is engineered to express luciferase upon stimulation of the VEGF receptor, producing bioluminescence. In the presence of an anti-VEGF molecule, VEGF binding to the receptor is repressed, interrupting the downstream signaling cascade, reducing luciferase expression, and decreasing bioluminescence [25].

**Figure 3:**
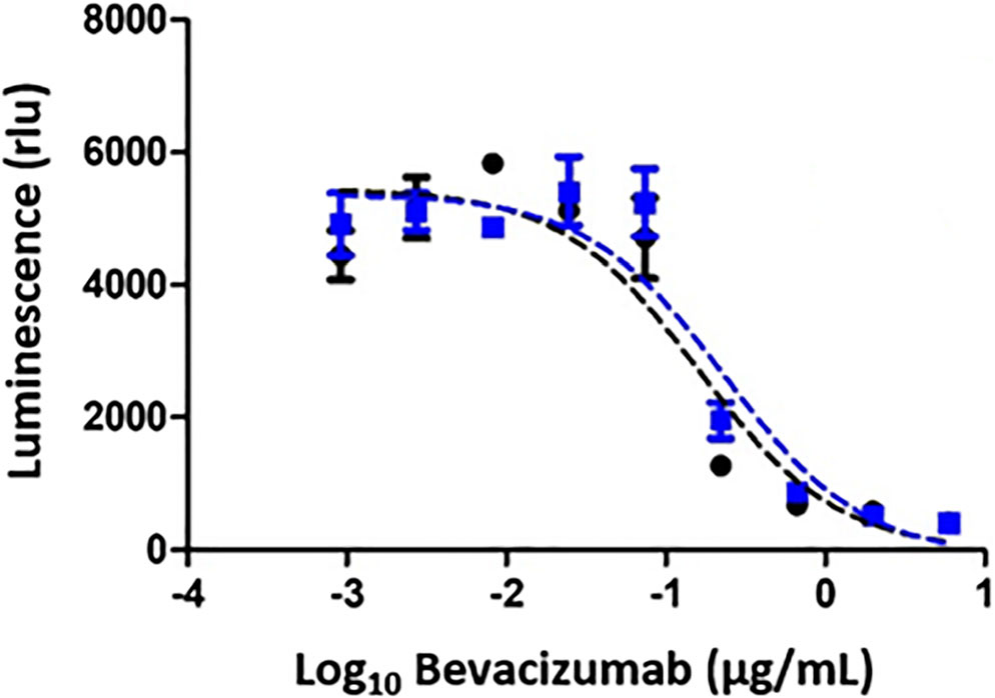
Anti-VEGF activities of bevacizumab spray-dried powder (blue squares) and as-received bevacizumab stock solution (black circles) in VEGF reporter assay

Results for the concentration dependence of luminescence for bevacizumab spray-dried powder and as-received bevacizumab stock solution are shown in Figure 3. The anti-VEGF activity of control and spray-dried powder are similar within the error of the assay, with IC_50_ values of 0.16 μg/mL and 0.23 μg/mL, respectively. This demonstrates that the biologic activity of the bevacizumab remains essentially unchanged after spray drying.

### Real-Time Stability

A real-time stability study was conducted with bevacizumab spray-dried powder stored at two conditions: 5°C and 25°C/60% RH. Samples were stored in sealed vials inside foil pouches with desiccant to reduce humidity exposure, which is known to recrystallize amorphous trehalose. Samples were analyzed before and after 1, 3, and 6 months storage at each condition. As the results in Table III show, minimal changes were observed in the stability samples, with physical stability and aerosol performance remaining constant throughout.

**Table III:** Stability results for bevacizumab spray-dried powder, showing IC_50_ sample-to-sample variability is similar to that of control

**Storage condition**	**Time (mo)**	***T***_**g**_ **Onset (°C)**	**PXRDresult**	**Potency(wt%)**	**FPF(%)**	**MMAD(μm)**	**IC**_**50**_ **(μg/mL)**
5°C	0	117	Crystalline L-leucine	37	81	2.2	0.23
	1	117		35	83	2.0	0.10
	3	122		35	78	2.0	0.10
	6	117		37	83	2.1	0.10
25°C/60% RH	0	117	Crystalline L-leucine	37	81	2.2	0.23
	1	119		37	81	2.3	0.13
	3	121		37	82	2.2	0.13
	6	118		38	84	2.5	0.17

### *In Vivo* Efficacy in Orthotopic Rat Model

The efficacy of the inhaled bevacizumab spray-dried powder for NSCLC treatment was evaluated in two tests using an orthotopic nude rat model: (1) a primary 8-week efficacy test and (2) a follow-on 4-week maintenance test.

#### Primary Efficacy Test

**Figure 4:**
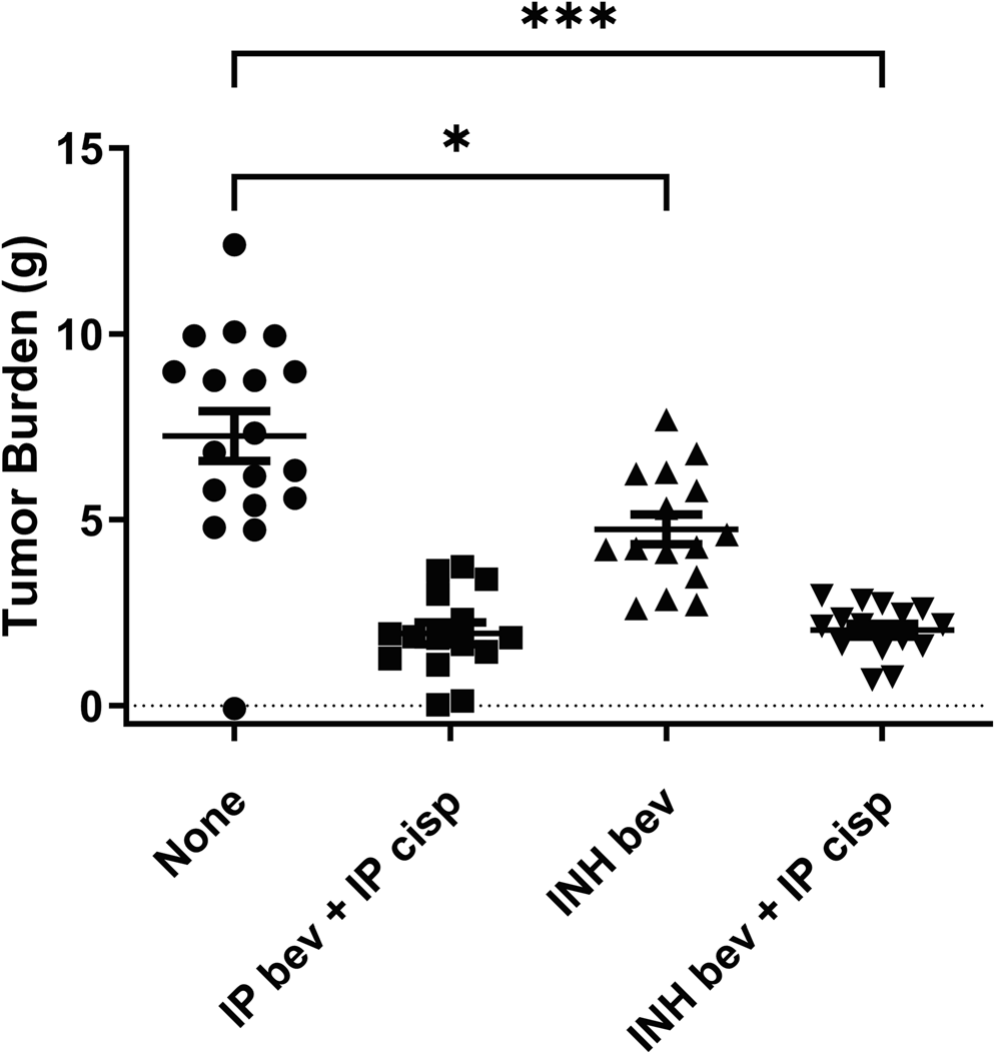
Primary treatment efficacy of inhaled bevacizumab spray-dried powder (INH bev) compared with injected bevacizumab (IP bev) and/or injected cisplatin (IP cis) in a nude orthotopic rat model for NSCLC treatment. *N* = 18 for untreated group, n = 15 for other groups, error bar is the mean ± one standard deviation. *p <0.05, ***p < 0.0005. Figure adapted from [39].

In the primary efficacy test, the bevacizumab spray-dried powder was evaluated for local inhaled delivery for NSCLC treatment in an orthotopic lung cancer nude rat model, with lung weight as the study endpoint. Untreated rats (study group 1) were the negative control, while rats treated with IP-injected bevacizumab and injected cisplatin (study group 2) were the positive control, representative of the standard of care for NSCLC [39]. As shown in Figure 4, the positive control reduced tumor burden significantly more than the negative control (mean lung weight of 1.9 g versus 7.3 g, respectively; *p* < 0.0005, a 74% reduction). The first experimental group (study group 3), rats treated with inhaled bevacizumab spray-dried powder alone, showed significant reduction in tumor burden compared to the negative control (4.7 g versus 7.3 g, respectively; *p* < 0.05, a 36% reduction). The second experimental group (study group 4), rats treated with inhaled bevacizumab spray-dried powder and injected cisplatin, also showed significant reduction in tumor burden (2.0 g versus 7.3 g, respectively; *p* < 0.0005, a 73% reduction). The reduction in tumor burden for the positive control and the inhaled bevacizumab/injected cisplatin combination treatment were indistinguishable (1.9 g versus 2.0 g, respectively). This is especially noteworthy because the delivered dose of inhaled bevacizumab is one-tenth the dose of injected bevacizumab (1.5 mg/kg versus 15 mg/kg). Therefore, delivery of the bevacizumab locally to the lung enables a tenfold dose reduction with equivalent efficacy in the rat model. In previous unpublished work from Lovelace Biomedical, a 42% reduction in tumor burden was found for rats dosed 2 mg/mL of cisplatin IP on a similar weekly regimen as that reported here. This suggests that both bevacizumab and cisplatin contribute to the reduction in tumor burden observed for the combination treatments.

#### Maintenance Efficacy Test

**Figure 5.**
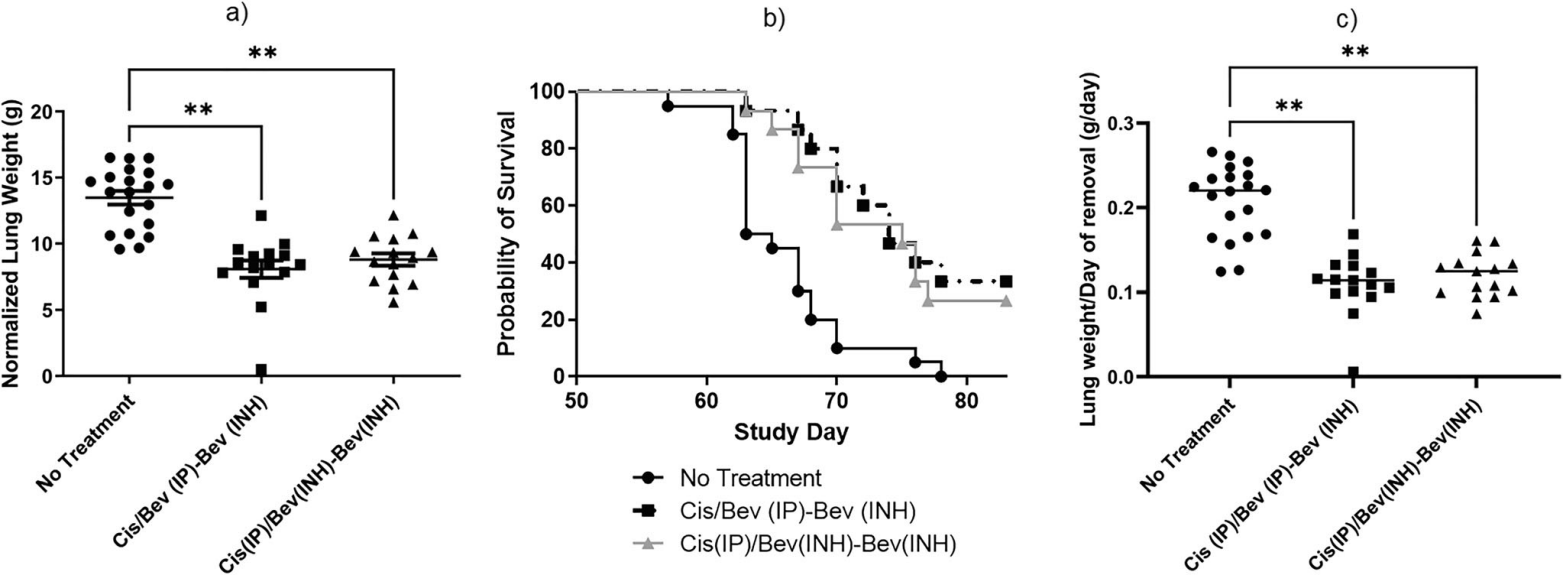
Efficacy of inhaled bevacizumab in maintenance trial, showing normalized lung weight (**a**), survival probability (**b**), and data adjusted to account for increased survival (**c**) in a nude orthotopic rat model for NSCLC treatment**.** *N* = 20 for untreated group, *n* = 15 for other groups, error bar is the mean ± one standard deviation. **p < 0.005. **c** is adapted from [39]

Maintenance treatment with bevacizumab is prescribed after completion of chemotherapy to continue the antiangiogenic effect in tumors, slowing regrowth. Maintenance efficacy of the inhaled bevacizumab treatment was evaluated in the rat model with lung weight and survival as endpoints. After primary treatment with injected cisplatin and either inhaled or injected bevacizumab, rats were treated with 4 weeks of maintenance inhaled bevacizumab. Both maintenance treatment groups had significantly lower lung weights (mean 8.1 g and 8.8 g versus 13.5 g, *p* <0.005 for both comparisons) and increased survival compared (74 and 75 days versus 64 days median survival) with untreated rats (Figures 5 a and b, respectively) [39]. Tumors had additional time to regrow in the treated maintenance groups due to their increased survival duration. To account for this effect, Figure 5c shows an additional presentation of the data, where the lung weight is divided by the individual rat’s date of removal from the study. These data show a more substantial difference between the treated and untreated groups.

## Discussion

The *in vivo* efficacy of inhaled bevacizumab at one-tenth the injected dose demonstrates the potential for dose reduction when a local treatment modality is used for lung cancer. Reports in the literature demonstrate similar effects for small molecule therapies administered to the lung as a dry powders [40]. Both topotecan [7] and 5-azacytidine [8] were administered to the lung as inhaled formulations. The dose-normalized area under the curve (AUC) for 5-azacytidine in lung tissue was 50 times higher for the dry powder aerosol compared with systemic administration. An inhaled topotecan dry powder formulation administered to the lung reduced tumor burden in rats significantly more than intravenous topotecan, despite a twofold reduction in dose. Particularly for highly potent therapies, local administration can provide a way of circumventing dose-limiting toxicity in patients.

Expansion of the local lung delivery concept to biotherapeutic molecules formulated for dry powder inhaler is of great interest for numerous indications. A recent review article summarized the potential of dry-powder inhalation formulations for administration of RNA to the lung [41]. A review by Frohlich and Salar-Behzadi in 2021 [42] summarized the inhalation delivery of proteins and peptides, most of which was accomplished via nebulization of liquid formulations.

Two dry powder inhalation formulations of mAb fragments (fAbs) have progressed to clinical trials to date. First, a phase I clinical trial was reported for dry powder inhaled formulation of VR942 (abrezekimab), a monoclonal antibody fragment used to treat asthma by inhibition of interleukin-13 [23]. A clinical trial is also in progress for the mAb fragment CSJ117 for asthma [42]. In another publication, Faghihi et al demonstrated successful formulation and administration of infliximab dry powder to suppress asthma-related inflammation in an animal model [22]. These studies, considered alongside this work, demonstrate the therapeutic potential of inhaled dry powder mAb formulations to treat a range of lung diseases.

The ability to administer bevacizumab in a dry-powder inhaler expands flexibility in treatment protocols. When a clinic visit is no longer required for bevacizumab administration, weekly or daily dosing becomes feasible. Self-administration of the treatment also eliminates the cost and inconvenience of recurring clinic visits. The physical stability of bevacizumab dry powder at ambient temperatures reduces supply chain and distribution constraints in getting medicine to patients. Overall, a bevacizumab dry powder formulation could bring great benefit to patients, removing many barriers to patient compliance. These same advantages could apply to any inhaled mAb therapy which replaces an IV formulation.

The spray-drying process and formulation approach described here provides a platform for manufacture of locally administered antibodies of interest to lung indications. At least 10 mAb therapeutics are currently approved in USA or EU for lung-related indications, including lung cancer, asthma, COPD, and pulmonary infections [43]. Application of the process and formulation and process learnings from this work to other mAbs of interest for pulmonary delivery could enable improved therapeutic outcomes for a broader range of lung diseases.

## Conclusion

This work reviewed the potential therapeutic benefits of pulmonary treatment of lung cancer by a spray-dried bevacizumab formulation. The bevacizumab spray-dried powder exhibited 6-month physical stability at 25°C, aerosol properties appropriate for pulmonary delivery, while retaining anti-VEGF activity. An orthotopic NSCLC rat model was used to test efficacy of the formulation *in vivo*, and it was found effective at reducing the tumor burden in the lung. When administered in combination with injected cisplatin, the inhaled spray-dried powder was as effective as injected bevacizumab at one-tenth the dose. This dose reduction could provide a way to minimize serious systemic side effects for bevacizumab and other mAb treatments. Pulmonary delivery of bevacizumab and lung cancer treatments, in general, are a promising development for patients and clinicians, opening the possibility of maintenance therapy administered at home with flexible dosing frequencies.
